# Shock

**Published:** 1898-03

**Authors:** 


### SHOCK.

ALTHOUGH the pathology of shock is still a debatable question, the treatment has been perfected until the results now obtained are very satisfactory both to patient and operator. The different theories regarding the pathology have undergone some criticism and experimental inquiry during the past year, especially by two American investigators, Dr. Eugene Boise, of Grand Rapids, Mich., and Dr. George W. Crile, of Cleveland, O. The latter's researches formed the essay to which was awarded the Cortwright prize fund of the College of Physicians and Surgeons of New York for 1897. The former in several papers has formulated a theory which appears to answer all the symptoms generally met with in shock following and during surgical operations.

Dr. Boise, in contributions to the transactions of the American Association of Obstetricians and Gynecologists, has attacked the old theories of paresis of the sympathetic system, paresis of the splanchnics, paresis of the cardiac and respiratory ganglia, paresis of the circulatory system, and has erected upon the ruins of these erstwhile theories another, which in many respects is worthy of more confidence and belief. To him nearly all the symptoms of shock can be explained on the theory that a *hyperirritation* of the entire sympathetic system is present and as a result we have stimulation of the vasomotors, contraction of the arterioles and a spasmodic action of the heart. The condition of the skin, pupils, heart's action, feeble pulse, scanty secretion of urine and the like, can all be easily explained on the theory of sympathetic stimulation, and when the therapeutic measures in vogue seem to meet the indications present the theory gains thereby in strength. Thus nitrite of amyl, nitroglycerine, morphine and moist heat are regarded as sedatives to the sympathetic system and relaxants of the arterioles and produce most favorable results.

Dr. Crile has experimented more with the blood pressure as affected by manipulation and irritation of the various organs and tissues of the body, and favors the treatment by intravenous injections of warm saline solutions along with a dilute solution of strychnia, slowly injected into the rubber tube of the infusion apparatus, thus entering the circulation directly in the stream instead of injecting it into the patient.

Dr. Crile's prize essay is to be published by the Lippincotts in the spring.

